# Anti-inflammatory properties of Risperidone : A clinical Trial

**DOI:** 10.1192/j.eurpsy.2023.436

**Published:** 2023-07-19

**Authors:** F. Askri, A. Aissa, S. Jedda, A. Ben Chaabene, H. Abaza, U. Ouali, Y. Zgueb

**Affiliations:** ^1^Avicenna, Razi Psychiatric Hospital, Manouba; ^2^Biology, Salah Azaiz, Tunis; ^3^Biology, Razi Psychiatric Hospital, Manouba, Tunisia

## Abstract

**Introduction:**

The evidence of an inflammatory status at onset of psychosis supports the adjunction of anti-inflammatory agent to antipsychotic (AP). Some negative results of these clinical trials lead us to wonder about the anti-inflammatory power of AP.

**Objectives:**

Would the action of associated anti-inflammatory agents be negligible compared to the anti-inflammatory potential of AP?

**Methods:**

We conducted a prospective study. We included patients hospitalized for acute relapse of schizophrenia. They were all treated with Risperidone. We measured High sensitive C Reactive Protein (Hs CRP) at baseline and 8 weeks after.

**Results:**

We included 24 patients. Mean age was 34,5 +7,32 years with 75% of female. Mean age of onset of illness was 24,63 ±4,81 years and illness duration was 10,70 +6,42 years. After 8 weeks, PANSS scores decreased significantly from 79,13 +12,07 to 47,21 +8,41 and Hs CRP levels dropped by 1,55 +3,96 mg/l.

**Conclusions:**

These results highlighted the anti-inflammatory action of Risperidone. Clinical trial should consider the proportion of anti-inflammatory agents action.

**Disclosure of Interest:**

None Declared

